# Coupling a Neural Network-Based forward Model and a Bayesian Inversion Approach to Retrieve Wind Field from Spaceborne Polarimetric Radiometers

**DOI:** 10.3390/s8127850

**Published:** 2008-12-03

**Authors:** Luca Pulvirenti, Nazzareno Pierdicca, Frank S. Marzano

**Affiliations:** Department of Electronic Engineering, Sapienza University of Rome, via Eudossiana 18, 00184 Rome, Italy; E-mails: pierdicca@die.uniroma1.it(N. P.); marzano@die.uniroma1.it (F. S. M.)

**Keywords:** Microwave radiometry, polarimetry, sea surface winds

## Abstract

A simulation study to assess the potentiality of sea surface wind vector estimation based on the approximation of the forward model through Neural Networks and on the Bayesian theory of parameter estimation is presented. A polarimetric microwave radiometer has been considered and its observations have been simulated by means of the two scale model. To perform the simulations, the atmospheric and surface parameters have been derived from ECMWF analysis fields. To retrieve wind speed, Minimum Variance (MV) and Maximum Posterior Probability (MAP) criteria have been used while, for wind direction, a Maximum Likelihood (ML) criterion has been exploited. To minimize the cost function of MAP and ML, conventional Gradient Descent method, as well as Simulated Annealing optimization technique, have been employed. Results have shown that the standard deviation of the wind speed retrieval error is approximately 1.1 m/s for the best estimator. As for the wind direction, the standard deviation of the estimation error is less than 13° for wind speeds larger than 6 m/s. For lower wind velocities, the wind direction signal is too weak to ensure reliable retrievals. A method to deal with the non-uniqueness of the wind direction solution has been also developed. A test on a case study has yielded encouraging results.

## 1. Introduction

Sea surface wind is a key parameter for the exchanges of heat and moisture between sea and atmosphere [1]. The sensitivity of polarimetric microwave radiometric observations to the wind vector has been demonstrated by several aircraft experiments (e.g. [2]). With respect to the scatterometers, a polarimetric radiometer has the additional capability of retrieving further sea (e.g. surface temperature) and atmospheric variables (e.g. water vapor). The WindSat radiometer [3], launched in 2003 aboard the Coriolis satellite, presently provides polarimetric passive measurements for retrieving the sea surface wind vector. It is a multifrequency instrument operating at 6.8, 10.7, 18.7, 23.8, and 37.0 GHz. The 10.7, 18.7, and 37.0 GHz channels are fully polarimetric, while 6.8 and 23.8 GHz channels are vertically and horizontally polarized.

Modeling the polarimetric microwave signature of the sea surface emission is essential for wind vector retrieval. A huge amount of studies, both theoretical and experimental, have been devoted to sea surface emission and scattering. A model of sea emission can be based on scattering models through the application of the Kirchoff's law. The Two-Scale Model (TSM) [4] is probably the most widely used method to generate synthetic satellite passive polarimetric observations, i.e., to simulate the modified Stokes vector (**T***_B_*), whose components are the brightness temperatures at vertical (*T_Bv_*) and horizontal (*T_Bh_*) polarizations, and the correlation parameters (*U* and *V*), emitted and scattered by a marine surface. Although several alternative methods have been developed, such as the Small Slope Approximation [5], it has not yet demonstrated that one method is superior with respect to the others [6].

In previous investigations, we have developed and validated a software package (named SEAWIND) based on the TSM and capable to simulate the fully polarimetric microwave passive observations of a spaceborne radiometer [7, 8]. This package has been recently improved and updated to produce synthetic backscattering coefficients as well (and its current name is SEAWIND2, hereafter denoted as SW2) [9]. In another study [10], which considered the WindSat polarimetric channels, we have exploited the universal approximation property of Neural Networks (NN's) to emulate the TSM, thus dealing with the problem of its low computational efficiency, which makes its use difficult to employ iterative techniques to solve the inverse problem.

The objective of this work, which basically represents the continuation of [10], is the investigation of the use of the NN's developed in [10] to tackle the inversion problem. In [10], the NN's have been trained to match the input-output relationship of the TSM (input: geophysical parameters; output: **T***_B_*). Here, we use the trained NN's to find the wind vector which best accounts for the measured **T***_B_*. To our knowledge, this is the first attempt to carry out an inversion of a NN-based forward model to estimate the wind field from polarimetric microwave radiometer data. Empirical geophysical model functions have been employed, as forward models, for this purpose in [1] and [11], while physically-based forward model functions have been exploited in [12] and [13]. Note that, in [10], we have demonstrated that Neural Networks (NN's) reproduce the behavior of the TSM fairly well, generally improving the accuracy achievable with a standard model function approach, so that an improvement on the quality of the retrieval, at least with respect to physically-derived model functions, can be expected.

To yield a reliable evaluation of the potentiality of the proposed approach for wind vector retrieval, particular attention is paid to the algorithmic aspects. Different Bayesian techniques [14, 15], such as Minimum Variance (MV), Maximum Likelihood (ML) and Maximum Posterior Probability (MAP) criteria have been used. Furthermore, for the latter two methods, an iterative inversion of the trained NN-based forward model has been performed and different methods to minimize the cost function, such as the common Gradient Descent and the more sophisticated Simulated Annealing, have been used.

In this work we present also a method, based on an evaluation of the curl and of the divergence of the wind vector, to deal with the non-uniqueness of the wind vector solution. To analyze the potentiality of this procedure, which can represent an alternative, or a complement, to the median filtering, commonly used for processing WindSat retrievals (e.g. [12]), a case study is considered.

The inversion techniques have been tested on simulated WindSat measurements generated by means of SW2, which has been applied to a set of meteorological analysis fields obtained from the European Centre for Medium-Range Weather Forecasts (ECMWF), collected throughout the first ten days of each month of year 2000 over the Mediterranean Sea. From such fields, whose spatial resolution is 0.5° both in latitude and in longitude, we have extracted the vertical profiles of temperature, pressure and relative humidity, as well as the surface value of sea temperature and wind velocity.

The work is organized as follows. Section 2 describes the procedure that we have adopted to retrieve the wind vector from the ECMWF-derived simulated **T***_B_*'*s*, as well as the proposed ambiguity selection technique. Section 3 is focused on the discussion of the results, while section 4 draws the main conclusions.

## 2. Wind vector retrieval

**Figure 1.**
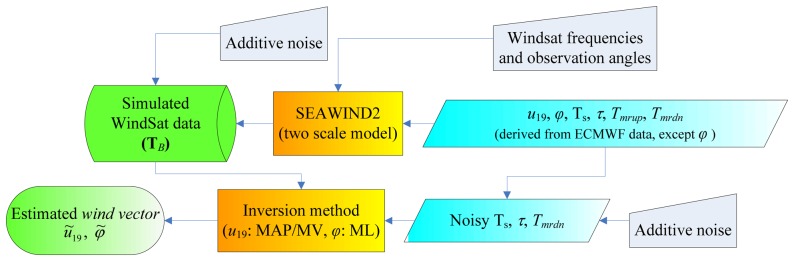
Block diagram of the procedure for wind vector retrieval adopted in this work.

The block diagram describing the procedure we have followed to test the retrieval algorithms is shown in Figure 1. The WindSat measurements have been simulated by running the SW2 software implementing the TSM. The inputs of SW2 are: azimuth angle between wind and radiometer pointing directions (*φ*), wind velocity at 19.5 m above sea level (*u*_19_), sea surface temperature (*T_S_*), atmospheric optical thickness (*τ*) and mean radiative temperatures (downwelling: *T_mrdn_* and upwelling: *T_mrup_*). As mentioned, these variables have been derived from the ECMWF analysis fields collected throughout year 2000, except for *φ* which has been randomly generated (with uniform distribution between 0° and 360°, in the absence of any a priori information). Atmospheric radiative parameters have been computed by applying a radiative transfer algorithm to the meteorological data, namely to the vertical profiles of pressure, temperature and relative humidity, assuming the presence of clear sky, or non-precipitating clouds [8]. To account for measurement noise and forward model errors, an additive Gaussian noise with zero mean and standard deviations equal to those found in [12] for the different WindSat channels has been added to the SW2-derived **T***_B_*'s.

ECMWF provides wind speed at 10 m of height, but the wave spectrum developed in [9] requires *u*_19_, so that SW2 has *u*_19_ as input parameter. The conversion has been carried out in two steps. Firstly, we have inverted the relationship used in [4], in which a neutral stability assumption is done, which allows the computation of the wind velocity at any height as a function of wind friction. In this way the wind speed at 10 m has been transformed in friction velocity. Then, the relationship has been applied to derive the wind velocity at 19.5 m. Hereafter, for ECMWF *u*_19_ we will intend the wind speed computed in this way.

Focusing on the wind vector, we have assumed that columnar cloud liquid and columnar water vapor in the atmosphere, as well as *T_S_*, have been already estimated by a separate algorithm. In the literature, several algorithms are available, such as the Ocean Algorithm for the Advanced Microwave Scanning Radiometer (AMSR) proposed in [16], where the expected retrieval accuracies for cloud liquid, water vapor and *T_S_* were foreseen equal to 0.02 mm, 1 mm and 0.5 K, respectively. To take into account the estimation error for *T_S_*, an additive Gaussian noise with zero mean and standard deviation of 0.5 K has been added to this variable. From the expected estimation errors for the atmospheric parameters, the uncertainties on *τ* and *T_mrdn_* have been evaluated through the relationships found in [16]. Note that *T_mrdn_* is the only mean radiative temperature used as NN's input, because *T_mrdn_* and *T_mrup_* are strongly correlated [10]. For *τ*, we have added a random Gaussian noise with zero mean and a standard deviation equal to the 10% of the average value of the ECMWF-derived *τ*. For *T_mrdn_*, we have added a random Gaussian noise with zero mean and a standard deviation equal to the 1% of the average value of ECMWF-derived *T_mrdn_*.

According to the scheme reported in Figure 1, the “noisy” *T_S_*, *τ* and *T_mrdn_*, as well as the vector of synthetic measurements **T***_B_*, are supplied to the block which accomplishes the inversion, producing the estimated wind velocity *ũ_19_*, and the estimated azimuth angle *φ̃*. The components of this block are described in the following sections. The inversion is performed in 3 steps. In the first step, the wind speed is retrieved and a fixed value of *φ* is used (180°). Successively, the azimuth angle is estimated through a ML criterion. Finally, the wind speed retrieval is refined by using *φ̃* instead of 180°.

The Bayesian theory of parameter estimation [14, 15] has been considered in this work to infer *u_19_* and *φ*. It focuses on the probability density function (pdf) *p* (*x*|**Y**) of a certain parameter *x* conditioned to the vector of measurements **Y**. By applying the Bayes theorem, *p* (*x*|**Y**) is given by:

(1) $$p(x|Y)=\frac{p(Y/x)p(x)}{p(Y)}$$

The criteria to estimate *x* may be different, depending on the cost associated to the errors of the estimation. We have used either MV or MAP criteria for wind velocity retrieval, while *φ̃* has been determined by means of a ML criterion.

### 2.1. MV-based wind speed retrieval

**Figure 2.**
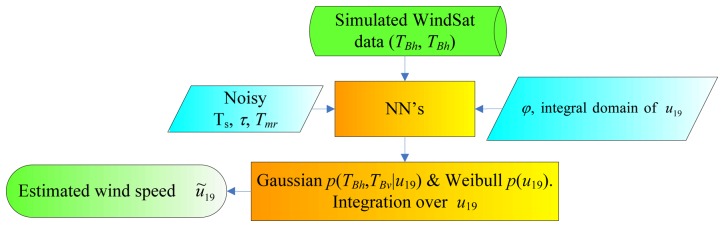
Block diagram of the MV wind speed estimator. Note that *φ* is equal to 180° (first step of the wind vector retrieval procedure), or to the estimated azimuth angle *φ̃* (third step).

The block diagram of the MV wind speed estimator is shown in Figure 2. Only the first two components of the modified Stokes vector, i.e., *T_Bh_* and *T_Bv_*, are used (as done in several papers, e.g. [1], [11]), since *U* and *V* are mainly sensitive to wind direction. The 6 GHz channels are not exploited.

The NN's block consists of four networks, one for each frequency included in the inversion process (i.e. 10, 18, 23 and 37 GHz). With respect to those developed in [10], in which only the fully polarimetric channels have been considered, a new NN for the 23 GHz band has been built, having the same architecture and the same learning method (Bayesian Regularization) of the other networks. Using *T_Bh_* and *T_Bv_*, only the corresponding two outputs of the NN's are delivered to the estimator.

It is well known that, if we compute the expected value of *p*(*u*_19_|**Y**) we obtain a MV estimator, which minimizes the root mean square (rms) retrieval error (and is also called Minimum Mean Square Estimator) [14, 15]. The wind speed estimate is therefore given by:

(2) $${\tilde{u}}_{19}=\int_{D}u_{19}p(u_{19}|Y)du_{19}=\frac{\int_{D}u_{19}p(Y|u_{19})p(u_{19})du_{19}}{\int_{D}p(Y|u_{19})p(u_{19})du_{19}}$$

where **Y**=[*T_Bh_*_10_*T_Bv_*_10_*T_Bh_*_18_*T_Bv_*_18_*T_Bh_*_23_*T_Bv_*_23_*T_Bh_*_37_*T_Bv_*_37_] *^T^* (superscript *T* indicates transposition) and *D* is the integral domain of *u*_19_, that we have chosen equal to [0-25 m/s], thus assuming that the pdf of wind speed *p*(*u*_19_) is zero outside this interval. Moreover, we have considered that *p*(**Y**) is obtained by saturating the joint probability *p*(**Y**, *u*_19_) = *p* (**Y**|*u*_19_) *p* (*u*_19_).

While *p* (**Y**|*u*_19_) has been supposed Gaussian, for *p* (*u*_19_) a Weibull distribution has been assumed [17], so that:

(3) $$p(Y|u_{19})=\frac{1}{\sqrt{2\pi \text{det}(C_{Y})}}\exp\left\{-\frac{1}{2}{\left[Y_{\text{meas}}-Y(u_{19})\right]}^{T}C_{Y}^{-1}[Y_{\text{meas}}-Y(u_{19})]\right\}$$

(4) $$p(u_{19})=\frac{k}{c}{\left(\frac{u_{19}}{c}\right)}^{k-1}\exp\left\{-{\left(\frac{u_{19}}{c}\right)}^{k}\right\}$$

In Equation (3), det(·) is the determinant, **Y**(*u*_19_) represents the output of the NN's based forward model, while **Y***_meas_* denotes the measurements vector and **C**_Y_ is the covariance matrix that should account for both measurements and forward model errors. We have assumed **C**_Y_ diagonal and its elements have been taken as those provided in [12]. For *p*(*u*_19_), the scale parameter *c* and the shape parameter *k* have been estimated from the ECMWF data used in this work through the ML formula suggested in [17], finding *c*=6.2889 m/s and *k*=2.2054.

To compute the estimate, i.e. the integrals at the third member of equation (2), a trapezoidal rule has been applied, sampling the domain *D* in intervals of 0.05 m/s.

### 2.2. MAP-based wind speed retrieval

If, instead of computing the expected value of *p*(*u*_19_|**Y**), we determine its maximum, a MAP criterion is applied. It is worth noting that, since the Weibull distribution is not a symmetric pdf, the MAP estimate differs from the MV one. Using the pdf s represented by equations (3) and (4), the MAP criterion reduces to minimize the following cost function with respect to *u*_19_ [14, 15]:

(5) $$d_{\text{MAP}}(u_{19})={\left[Y_{\text{meas}}-Y(u_{19})\right]}^{T}C_{Y}^{-1}[Y_{\text{meas}}-Y(u_{19})]-2(k-1)\ln(u_{19}/c)+2{\left(u_{19}/c\right)}^{k}$$

**Figure 3.**
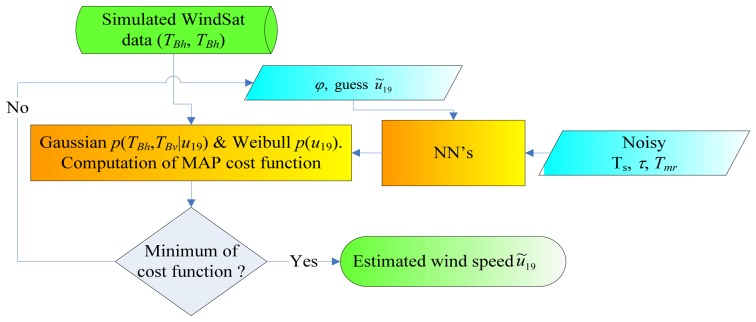
Block diagram of the MAP wind speed estimator. Note that *φ* is equal to 180° (first step of the wind vector retrieval procedure), or to the estimated azimuth angle *φ̃* (third step).

Figure 3 shows the block diagram of the MAP wind speed estimator. The minimization has been carried out through an iterative procedure, thus accomplishing an iterative inversion of a trained NN-based forward model, as proposed in [18]. Two optimization techniques have been used, both starting from a first guess equal to the mean value of the ECMWF *u*_19_. Besides the conventional Gradient Descent (GD) method (adopted in [1]), a stochastic advanced method such as Simulated Annealing (SA) has been employed too. A description of the routines implementing these techniques can be found in [19]. According to the selected algorithm, a new guess value for *u*_19_ is produced and the NN-based forward model generates the corresponding **Y**(*u*_19_).

GD evaluates both the cost function and its gradient, basing on the idea that the direction of the negative gradient indicates where the minimum of the cost function is placed. According to this direction, another point, where cost function and gradient are evaluated, is selected and the new gradient identifies the new direction of the minimum. The iterations end when a (local) minimum of the cost function is found. GD is strongly dependent on the first guess.

As opposed to GD, SA is a global optimization algorithm. It associates to each point of the domain in which the minimum of the cost function is searched, a state of a physical system characterized by an internal energy. The minimum of the cost function corresponds to a state with the minimum possible energy. At each step of the optimization, SA considers some neighbors of the current state, randomly generated by perturbing the current state itself. Then, SA decides whether accepting or rejecting the new configuration basing on the difference between its energy and the previous one. If the energy of the new state is smaller than the original one, the new state is accepted, otherwise it is accepted with a probability depending on the Boltzmann statistics. The procedure ends when the system reaches a state that is good enough for the application, or when computational time reaches the maximum fixed by the user.

### 2.3. ML-based wind direction retrieval

**Figure 4.**
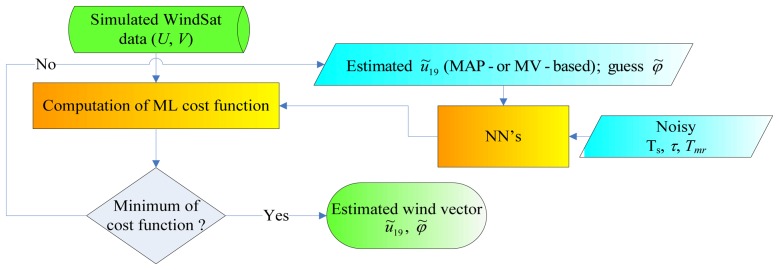
Block diagram of the ML wind direction estimator.

The wind direction is identified by the azimuth angle *φ*=*φ_r_*−*φ_w_*, where *φ_r_* is the radiometer pointing direction and *φ_w_* is the wind direction. The block diagram of the ML wind direction estimator is shown in Figure 4. It uses the third and fourth components of the modified Stokes vector, i.e., *U* and *V* of the fully polarimetric WindSat bands (10, 18 and 37 GHz). The choice of a ML estimator is related to the assumption of a uniform distribution for *φ*. The cost function to be minimized is given by:

(6) $$d_{ML}(\varphi )={\left[Y_{\text{meas}}-Y(\varphi )\right]}^{T}C_{Y}^{-1}[Y_{\text{meas}}-Y(\varphi )]$$

where **Y**=[*U*_10_*V*_10_*U*_18_*V*_18_*U*_37_*V*_37_] *^T^* and, also in this case, the error covariance matrix **C**_Y_ as been assumed diagonal with elements taken as those found in [12]. The wind velocity supplied as input to the NN's is that estimated in the first step of our wind field retrieval procedure. Also for the minimization of *d*_ML_ an iterative technique, based on the two optimization methods introduced above, has been accomplished.

It is well known that, when dealing with the retrieval of wind direction, the problem of the non-uniqueness of the solution is fairly significant. The cost function may have many local minima and different solutions (generally indicated as ambiguities) can be obtained depending on the first guess value of *φ̃* (hereafter denoted as *φ̃_fg_*) [1], [11,12]. We have searched for the four ambiguities, as done in [12], so that the procedure illustrated in Figure 4 has been applied four times (for each **T***_B_* of the ECMWF-SW2 test set), each time using a different *φ̃_fg_*. One value of *φ̃_fg_* (denoted as *φ̃_fg_*_1_) has been obtained by evaluating *d_ML_* every 5°, spanning the interval [0°-355°], and selecting the azimuth angle yielding the smallest *d_ML_*. The other three values of the first guess have been chosen to be *φ̃_fg_*_1_+90°, *φ̃_fg_*_1_+180°, *φ̃_fg_*_1_+270° [12].

### 2.4. Ambiguity selection

In this section, a method to deal with the non-uniqueness of wind direction solution that exploits the contextual information by basing either on the curl of the wind vector **u** (∇ × **u**), i.e., on wind vorticity, or on its divergence (∇ · **u**), is illustrated. This technique is an alternative to methods used for wind vector retrieval from WindSat that are based on median filtering [11, 12]. The idea on which the method is based is that, in an area characterized by an unrealistic dispersion of wind directions, the norm of the vorticity and/or of the divergence is large. Selecting the ambiguity that yields the minimum value of the norm may lead to a more reliable *φ̃*.

**Figure 5.**
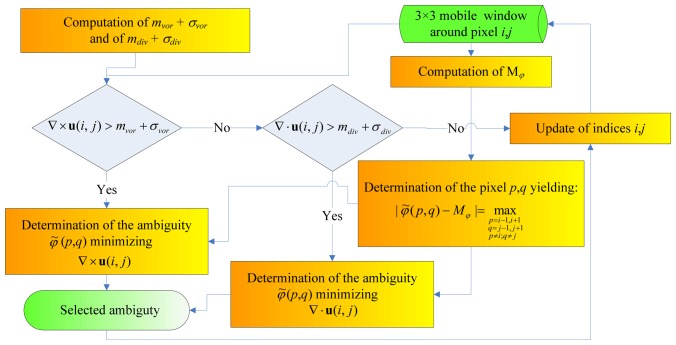
Block diagram of the ambiguity selection procedure. Indices *i*, *j* denote the central pixel of the 3×3 mobile window; *p* may be equal to *i*-1, or *i*+1; *q* may be equal to *j*-1, or *j*+1.

The block diagram illustrating the ambiguity selection method is shown in Figure 5. Firstly, both the mean values and the standard deviations of |∇ × **u**| (denoted as *m_vor_* and *σ_vor_*, respectively) and |∇ · **u**| (*m_div_* and *σ_div_*) in the considered swath area are computed. Then, a mobile window of 3×3 radiometric pixels is considered and the median of the wind directions within the window (*M_φ_*) is computed. Only points presenting either |∇ × **u**|> *m_vor_* + *σ_vor_* or |∇ · **u**|> *m_div_* + *σ_div_* are processed. We have based the computation of the vorticity, as well as of the divergence, on the four pixels adjacent to the centre of the moving window along both the vertical and the horizontal directions. Among these four pixels, the procedure acts on that presenting the largest value of |*φ̃* − M*_φ_*|. Then, among the four ambiguities associated to this pixel, the one yielding the minimum value of the curl or of the divergence is selected. The procedure is iterated until the wind vector ends changing its direction.

## 3. Results

The wind vector retrieval algorithms proposed in this work have been applied to the 1,000 records of the ECMWF-SW2 test set. In what follows, we will firstly evaluate the performances of the various wind speed estimators, by comparing the estimates with the ECMWF *u*_19_, and then we will deal with the wind direction. We remind again that the wind directions used as inputs of SW2 to produce the test set have been randomly generated.

### 3.1. Results of the wind speed retrieval

The values of the statistical parameters used to evaluate the performances of the wind velocity retrieval methods are reported in Table 1. They regard the results obtained, at the end of the whole retrieval procedure, using as input of the NN's the *φ̃*, that is closest to the test set wind direction. Hereafter, this *φ̃*, will be denoted as the closest wind direction ambiguity [11, 12]. Indicating by *ε_u_* the estimation error (i.e., the difference between *ũ*_19_ and ECMWF *u*_19_), these parameters are the average value of *ε_u_* (*ε̅_u_*), its standard deviation *σ_u_* and the correlation coefficient *ρ* between *ũ*_19_ and ECMWF *u*_19_. Table 1 suggests that the behavior of MV is the best, not only in terms of *σ_u_* (as expected), but also of *ρ* while *ε̅_u_* is approximately 0 m/s for MV and MAP-SA. The performances of MAP-GD are the worst due to the fact that GD is a local optimization method, as opposed to SA, which is a stochastic global optimization algorithm. SA has revealed the quickest technique, requiring half of the computational time of MV and GD.

**Figure 6.**
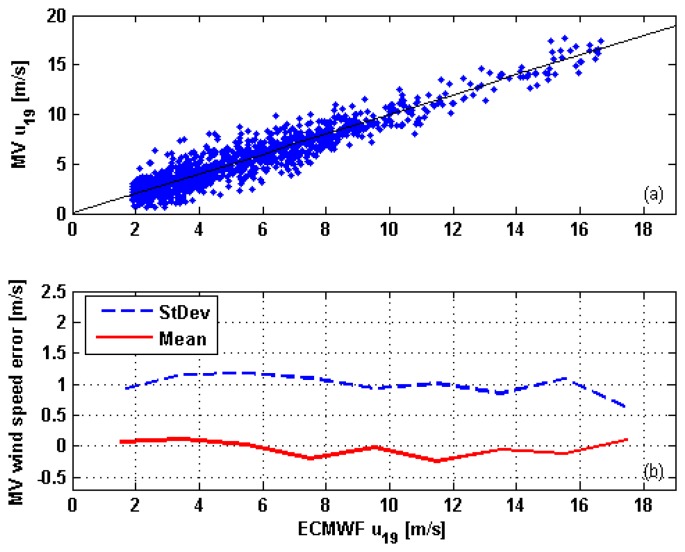
Estimated versus ECMWF wind velocities (a), and binned analysis of the mean (solid red line) and of the standard deviation (dashed blue line) of *ũ*_19_ − *u*_19_ (b). In panel a, the black solid line indicates perfect agreement. Both panels concern MV.

The performance of the MV wind speed retrieval is shown in Figure 6. Upper panel (a) shows the scatterplot of ECMWF versus estimated *u*_19_. Lower panel (b) shows a binned analysis of the estimation error in which the range of ECMWF *u*_19_ has been divided into 2 m/s wide intervals [11, 12], and for each interval the mean and the standard deviation of the difference between *ũ*_19_ and ECMWF *u*_19_ have been computed. The standard deviation of the wind speed retrieval error is always less than 1.2 m/s, while its average value is generally less than ±0.2 m/s. These results can be compared with those found in [11] in which, likewise the present study, the algorithm to determine the atmospheric parameters is detached from the inversion algorithm to evaluate the surface wind vector. In [11], a standard deviation of wind velocity estimation error of 1.5 m/s, larger than that presented by our estimators (except MAP-GD) is foreseen, while the values of the mean span the interval ±0.5 m/s.

Finally, it is worthwhile to briefly summarize the results achieved in the first step of our procedure, i.e., for the wind speeds estimates obtained using *φ* = 180°, since they are supplied to the block of the wind vector estimator which determines *φ̃* (see Figure 4). Also in this case, MV yields the best performances, with *ε_u_* = 0.32 m/s and *σ_u_* =1.21 m/s. For this reason, the following section will consider only the performances achieved by supplying the block that retrieves wind direction with the MV-estimated wind speeds.

### 3.2. Results of the wind direction retrieval

**Figure 7.**
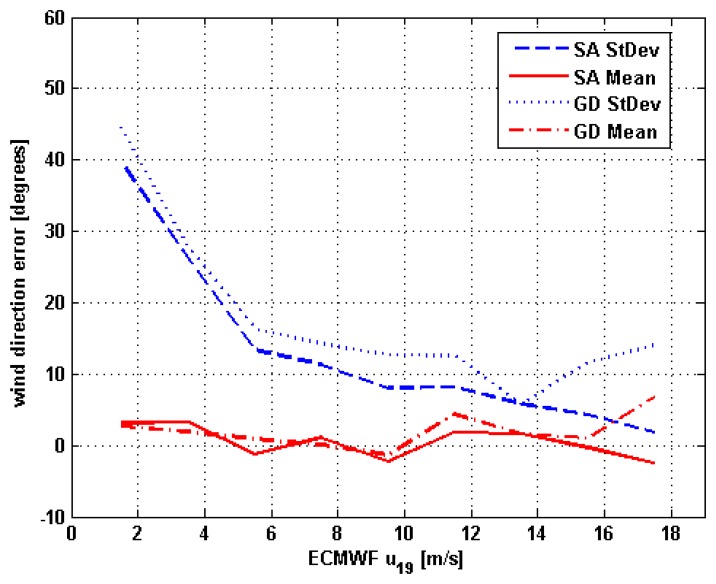
Binned analysis of mean (red lines) and standard deviation (blue lines) of the difference between the closest ambiguity and the test set *φ* versus wind speed.

Figure 7 shows the mean and the standard deviation of the difference between the wind directions belonging to the test set and the estimated ones versus the ECMWF wind speed (2 m/s bins). This difference is assumed as the wind direction estimation error [11, 12]. Note that, since *φ* is a periodic circular variable, the zero is arbitrary and the notion of high and low values may be questioned. Consequently, in the treatment of wind direction there are various algorithms that are employed to compute the standard deviation and, as discussed in [20], the choice depends on the experimenter. To be consistent with [11, 12], here we have decided to consider the arithmetic mean and standard deviation and we have assumed that the maximum values of the difference between the directions are ±180°. This means that, if, for example, the test set *φ* is 10° and *φ̃* = 350°, we assume that the error consists of an underestimation of 20° (-10°-10°), rather than an overestimation of 340° (350°-10°). Among the four outputs of the ML estimator, the ambiguity that is closest to the test set wind direction (closest ambiguity) is considered in Figure 7.

Figure 7 suggests that the mean of the wind direction retrieval error is fairly small for both ML-SA and ML-GD, whose behavior is similar for ECMWF *u*_19_<16 m/s, which is the wind speed range to which most of the records of the test set belong. It is well known that a problem presented by GD is that it finds a local minimum, not necessarily the absolute one. In this case, the use of four ambiguities seems to mitigate this problem. Also the efficiencies of the two methods have revealed comparable. However, the performances of SA are better in terms of the standard deviation of the wind direction retrieval error. For this parameter, the largest values are associated to low wind regimes, for which the signal due to wind direction is weak [21]. Similar results have been found in [11]. Considering SA, the standard deviation of the wind direction retrieval error is less than 13°, for ECMWF *u*_19_>6 m/s, and less than 9° for ECMWF *u*_19_>10 m/s. In [11], it is stated that the standard deviation of the ambiguity closest to the adopted forward model output is less than 20° discarding wind speeds smaller than 6 m/s and less than 15° for wind speeds greater than 10 m/s.

### 3.3. Results of the ambiguity selection method: a case study

The fairly good results shown in Figure 7 concern the closest ambiguity. If we rank the four solutions produced by the ML-based estimator according to the value of the corresponding cost function and consider the one yielding the least *d_ML_* [see equation (6)], the performances of both the wind direction retrieval algorithms get worse, particularly in terms of the standard deviation of the wind direction estimation error. This implies that simply selecting the ambiguity on the basis of the cost function leads to retrieval inaccuracies [13], so that an appropriate selection of the ambiguity is fundamental to achieve reliable results.

While in the previous sections we have yielded a statistical evaluation of the performances of the inversion algorithms, we have decided to rely on a case study to evaluate the proposed ambiguity selection technique. This case study concerns the ECMWF analysis of 6 March 2000 (relative to the Mediterranean Sea). The ECMWF data have been treated as described in section 2 to derive both the atmospheric and the surface parameters. In this case, the ECMWF data about the direction where the wind is blowing (i.e. those regarding *φ_w_*) have been used too. To derive *φ* from *φ_w_*, the information on *φ_r_* is needed. For producing this information, we have numerically simulated a radiometric image, acquired by a conically-scanning radiometer similar to WindSat, as we have done in [22].

**Figure 8.**
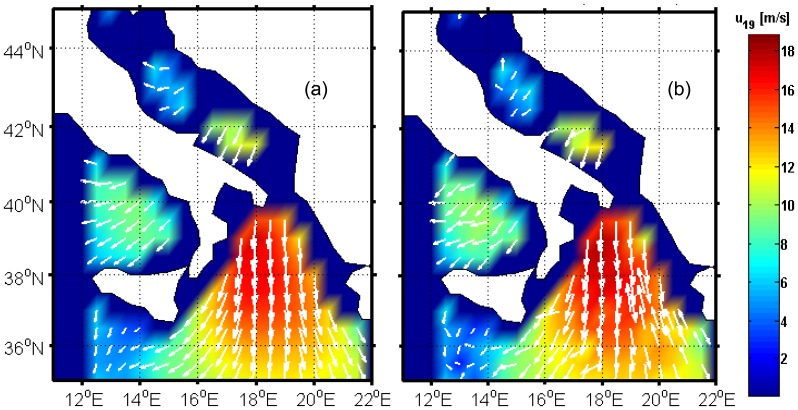
ECMWF (a) and estimated (b) wind fields for the analyzed case study. White arrows denote direction where wind is blowing. Their length is proportional to wind speed.

We have simulated a WindSat forward scan considering 80 pixels with a spacing of 12.5 km along and across scan [12]. A geographical box between 35° N and 45° N and between 12.5° E and 21.5° E has been assumed and a satellite track from North to South along the 17° meridian has been supposed. Figure 8a shows the wind vector field derived from ECMWF data and from the simulation procedure depicted in detail in [22]. White areas denote land, while dark blue areas in which the white arrows representing wind direction are absent, are due to an unrealistic identification of land areas by the ECMWF land/sea mask (caused by the poor resolution of the ECMWF data, that is 0.5°, both in longitude and in latitude), or indicate areas outside the considered geographical box (longitudes less than 12°).

Figure 8b shows the results of wind vector retrieval procedure described in the previous section when applied to the present case study. The map of *ũ*_19_ has been produced by applying the MV estimator, while *φ̃* through ML-SA. The intensity of the wind vector resembles that in Figure 8a, while for wind direction, the clearest differences between panels (a) and (b) can be seen at longitudes around 19°-21°, in which an error of approximately 180° is present.

**Figure 9.**
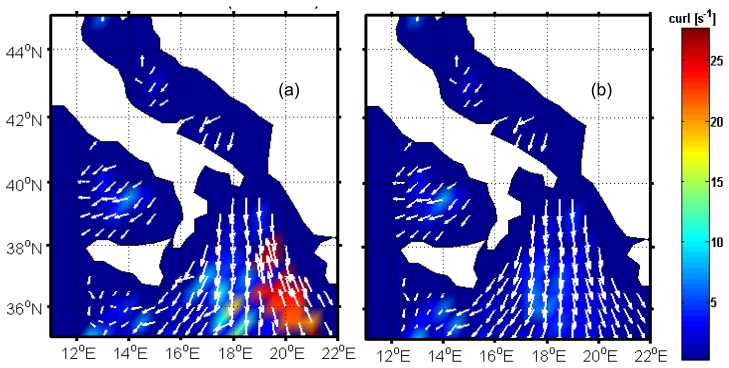
Norm of the curl of the estimated wind field before (a) and after (b) the application of the proposed ambiguity selection procedure.

Hereafter, when referring to curl and divergence we will intend their norm (see section 2.4). Figure 9a shows the wind vorticity corresponding to that in Figure 8b. The highest values of this quantity are located around the area where we make the largest errors in direction retrieval. This occurs for divergence too, so that it is not shown for conciseness. The result of the ambiguity selection method, obtained after three iterations, is shown in Figure 9b. The final wind field presents a vorticity considerably less than that in Figure 9a and the wind direction agrees with the ECMWF analysis.

It is worth noting that advanced ambiguity selection techniques, such as sophisticated model-based approaches (e.g. [23]), or variational analysis methods (e.g. [24]) have been developed for radar scatterometers. We underline again that our technique should be considered an alternative, or a complement, to methods used for wind vector retrieval from WindSat that are based on simple median filtering ([11, 12]), so that we do not claim to improve these advanced techniques. With respect to median filters our method relies on physical considerations, although very simple. In addition, many ambiguity selection algorithms (e.g. [12]) are initialized with a “nudged” wind field from an external data source, such as a Numerical Weather Prediction model. Here we are considering the situation in which the only source of available data is the radiometer, i.e., a sort of worst case, so that we do not exploit external information.

## 4. Conclusions

A simulation study aiming at evaluating the potentiality of a wind vector retrieval algorithm for a polarimetric radiometer, based on the approximation of the forward model by means of a NN approach, has been presented. An effort has been devoted to test several inversion algorithm, all based on the Bayesian theory of parameter estimation. In particular, MV and MAP for wind velocity, and ML for wind direction, have been considered. To minimize the cost function of both MAP and ML, a conventional technique such as GD, and a more advanced optimization method, such as SA have been employed.

The polarimetric measurements have been simulated by means of a software package, already developed and validated, implementing the two scale model. The software has been fed by data derived from the ECMWF analysis fields. The atmospheric parameters, as well as the sea surface temperature have been assumed already estimated and an uncertainty on the inputs of the NN-based forward model has been associated to the expected retrieval error of these quantities.

The best result for the wind speed estimation has been achieved by the MV algorithm, for which the standard deviation of the retrieval error is approximately 1.1 m/s. The performances of MAP-SA and, especially of MAP-GD are worse, although the former has the best computational efficiency.

As for the wind direction, ML-SA has revealed the best estimator, yielding a standard deviation of the wind direction error for the closest ambiguity less than 13° for wind speeds larger than 6 m/s and less than 9° for wind speeds greater than 10 m/s. As already found in the literature, for lower wind velocities, the wind direction signal is weak. Moreover, we have found that the performances achieved by considering the ambiguity yielding the least value of the ML cost function are worse, so that the ambiguity selection has revealed a critical aspect.

An ambiguity selection method exploiting the contextual information by basing either on the curl of the wind vector, or on its divergence, has been finally proposed to represent an alternative to median filtering used in other papers dealing with wind retrieval from WindSat data. Its application on a case study has yielded encouraging results.

## Figures and Tables

**Table 1.** Statistics of the comparison between ECMWF and estimated wind speeds for MV, MAP-GD and MAP-SA.

	MV	MAP-GD	MAP-SA

*ρ*	0.94	0.89	0.90
*ε̅_u_* [m/s]	−0.01	0.34	0.02
*σ_u_* [m/s]	1.09	1.41	1.23
